# Gestational diabetes and mental health: longitudinal analysis of data from the GEMS randomized trial

**DOI:** 10.1007/s00737-024-01551-0

**Published:** 2025-01-15

**Authors:** Phyllis Ohene-Agyei, Greg D. Gamble, Thach Tran, Jane E. Harding, Caroline A. Crowther

**Affiliations:** 1 Liggins Institute, Faculty of Medical and Health Sciences, University of Auckland, 85 Park Road, Grafton, Auckland, 1023 New Zealand; 2 School of Biomedical Engineering, University of Technology Sydney, Sydney, NSW Australia

**Keywords:** Gestational diabetes, Anxiety, Depression, Health related quality of life, Perinatal mental health

## Abstract

**Purpose:**

There is limited high-quality evidence about perinatal mental health among women with gestational diabetes. We aimed to assess the risks and longitudinal changes in anxiety, depression, and health-related quality of life comparing women with gestational diabetes and those without among a contemporary cohort of pregnant women.

**Methods:**

Prospective cohort study of participants in the GEMS Trial. Women with a singleton pregnancy were eligible if they had a 75-g diagnostic oral glucose-tolerance test between 24 and 32 weeks’ gestation, provided written informed consent, and completed questionnaires about anxiety, depression, and health-related quality of life at the study time points.

**Results:**

There were no differences in risk for anxiety (RR 1.13, 95% CI 0.86, 1.49; *p* = 0.39) or depression (RR 1.08, 95% CI 0.78, 1.50; *p* = 0.64) between the two groups at 36 weeks’ gestation or 6 months postpartum [anxiety: (RR 1.21, 95% CI 0.90, 1.63; *p* = 0.21); depression: (RR 0.84, 95% CI 0.55, 1.28; *p* = 0.43]. However, at 36 weeks’ gestation participants with gestational diabetes reported better physical functioning, and at 6 months postpartum, better mental functioning (mean difference (MD) in scores 1.28, 95% CI 0.25, 2.30; *p* = 0.01) although worse physical functioning (MD -2.99, 95% CI -3.90, -2.07; p = < 0.001) compared to participants without.

**Conclusion:**

The risk for poor mental health during the perinatal period does not differ importantly among women diagnosed and treated for gestational diabetes compared to the general pregnant population.

## Highlights

In this prospective cohort of 3051 trial participants, we found a similar risk for anxiety and depression among participants with gestational diabetes compared to those without.

However, participants with gestational diabetes had better mental health-related quality of life but worse physical health-related quality of life postnatally.

Women with gestational diabetes may have suboptimal physical health related quality of life in the perinatal period, but clinicians and patients should be reassured that risk of anxiety and depression does not differ from the general pregnant population.

## Introduction

Poor mental health outcomes are the most common disorders experienced by women in the antenatal period and after childbirth. A systematic review including studies from 34 countries reported, with high-certainty evidence, a prevalence of 22% for antenatal anxiety and 12% for postnatal anxiety up to six months after birth among healthy pregnant women (Dennis et al. 2017). Similarly, recent estimates from a review of systematic reviews found that 29% of pregnant women experience depression in the antenatal period and 28% in the postnatal period (Al-abri et al. 2023). Perinatal mental disorders are associated with multiple adverse outcomes for mother and child in both the short and long-term. During pregnancy, poor mental health predisposes to an increased risk of gestational hypertension (Rusner et al. 2016), preterm birth (Dadi et al. 2020), and birth of a low birthweight baby (Rusner et al. 2016; Dadi et al. 2020). In the postpartum period, mental disorders are associated with poor maternal coping responses (George et al. 2013), and reduced ability to breastfeed (Ahmadinezhad et al. 2024). Children born to mothers with perinatal mental disorders have higher rates of mental distress, behavioral problems, and neurocognitive disorders in later life (O’Connor et al. 2002, 2003; Mennes et al. 2006). As a result of these adverse outcomes, perinatal mental disorders are associated with a significant economic burden. In the United Kingdom, lifetime costs of perinatal anxiety and depression was estimated to cost £6.6 billion(Bauer et al. 2016) and in the United States, perinatal mood and anxiety disorders was estimated to cost $14 billion from conception to 5 years postpartum (Luca et al. 2020).

Gestational diabetes mellitus (GDM), defined as carbohydrate intolerance of varying degree with first onset in pregnancy (World Health Organization 1999), is the commonest metabolic disorder in pregnancy, and is associated with adverse outcomes including maternal pre-eclampsia (Plows et al. 2018), induced labour (Plows et al. 2018; Shen et al. 2020), and birth of a large-for-gestational age baby (Plows et al. 2018). There is also an increased risk of poor mental health including depression (Arafa and Dong 2019; Azami et al. 2019; Riggin 2020), anxiety, and stress (Daniells et al. 2003). A concurrent diagnosis of GDM and poor mental health during pregnancy has been associated with increased rates of perinatal complications such as preterm birth, neonatal respiratory distress, gestational hypertension, and pre-eclampsia (Lee et al. 2020; Packer et al. 2021).

Most studies assessing perinatal mental health among women with GDM, have been cross-sectional or included small sample sizes.(Wilson et al. 2020) They have predominantly focused on anxiety and depression (Wilson et al. 2020; Ouyang et al. 2021), but health-related quality of life (HRQoL) also has been reported to deteriorate among women with GDM (Marchetti et al. 2017). Few studies have reported mental health in both antenatal and postnatal periods.

We aimed to compare the prevalence of risk for anxiety, vulnerability to depression, and HRQoL at 36 weeks’ gestation and 6 months postpartum between women who were diagnosed with GDM and those without, in a large multi-ethnic randomized trial cohort, and to assess how these changed over time.

## Methods

This is a secondary analysis of the GEMS Trial, a randomized trial that assessed the optimal glycemic thresholds for GDM diagnosis to improve perinatal outcomes (Crowther et al. 2022). Women with a singleton pregnancy were eligible if they had a 75-g oral glucose-tolerance test (OGTT) at 24 to 32 weeks’ gestation and provided written informed consent. Women with diabetes mellitus or a history of gestational diabetes were ineligible. Women whose OGTT results indicated gestational diabetes based on their allocated criteria group were provided with GDM care comprising nutritional therapy and as-needed pharmacologic treatment, and women whose OGTT indicated no GDM received routine pregnancy care.

Eligible participants for this study were women who completed any questionnaire screening for anxiety, depression, and health-related quality of life (HRQoL) at study enrolment (baseline), 36 weeks’ gestation, or 6 months postpartum.

### Outcome measures

Anxiety was measured using the 6-item Spielberger State-Trait Anxiety Inventory (STAI), a valid alternative to the full version for use in research (Court et al. 2010), with scores > 15 indicating the presence of symptoms of anxiety (Crowther et al. 2005).

Depression was assessed using the Edinburgh Postnatal Depression Scale (EPDS), a validated tool for use among pregnant women (Cox et al. 1987). The tool comprises 10 items with each item scored on a 4-point scale (0–3) for a maximum score of 30. We used a score of > 12 to indicate vulnerability to depression, a cut-off shown to have the highest specificity in both the antenatal and postpartum period (Levis et al. 2020).

Health-related quality of life was assessed using the 36-Item Short-Form General Health Survey (SF-36), which assesses eight aspects of health status: general health, mental health, physical functioning, social functioning, role physical, role emotional, bodily pain, and vitality (Ware and Sherbourn 1992). Scores range from 0 to 100, with two summary measures, namely physical component summary (PCS) and mental component summary (MCS), with higher scores indicating higher levels of functioning (Ware 2000). The MCS has a standardized mean (standard deviation) of 50 ± 10 derived from the general healthy New Zealand population (The Ministry of Health New Zealand 1999). We assigned a cut-off score below minus one standard deviation (MCS < 40) as denoting poor mental HRQoL, as this adequately captures mental health outcomes and has good positive predictive value for poor mental health outcomes compared to other validated psychological instruments (Ware, MA and Keller, 1993; Pfoh et al. 2016).

### Statistical analyses

Baseline characteristics were compared between women who did and did not receive a diagnosis of GDM using Student’s t-tests or chi-square tests. Mental health outcomes were analyzed both as continuous and categorical variables using generalized linear mixed-effects models (log-binomial regression for binary outcomes and normal distribution (identity link function) for continuous outcomes) to estimate relative risks (RR) and mean differences (MD) with their 95% confidence intervals (CIs). A two-sided p-value < 0.05 was considered statistically significant. No adjustment was made for multiple comparisons. Statistical analyses were conducted using SAS software version 9.4 (SAS Institute, Cary, North Carolina, United States of America).

Repeated measure analyses were undertaken using generalized linear mixed-effects models to determine if there was an interaction between GDM status (yes/no) and time. We fitted time and GDM status as main effects and included their interaction using the SAS proc mixed. An unstructured covariance type was assumed to allow for flexibility in the covariance structure. Additionally, analyses were performed with change from baseline as the dependent variable using analyses of covariance and including baseline values as covariates to present results independent of differences at baseline. Marginal least square means and their corresponding 95% confidence intervals (CI) are presented.

No imputation of missing data was performed since we could not necessarily meet the assumption of missing at random. However, the mixed-effects model approach used is robust for effectively handling missing data and does not omit data due to missingness.

## Results

At study enrolment (baseline), 3051 participants (75.1% of the total GEMS Trial participants) completed the mental health questionnaires and were eligible for inclusion in this study. Of these, 313 (10.2%) were diagnosed with GDM. Questionnaires were completed by 2888/3051 (94.5%) at 36 weeks’ gestation and 2082/3052 (68.2%) at 6 months after birth.

Baseline characteristics differed between the two groups, with more participants of Asian ethnicity in the GDM group (49% compared to 31%), and European ethnicity in those without GDM group (42% compared to 24%) (Table 1). Additionally, 50.8% of participants in the GDM group reported a family history of diabetes compared to 32.8% in the no GDM group. Participants with GDM were older and had a mean 2kg/m^2^ higher body mass index (BMI) than those without.

**Table 1:** Baseline characteristics of study participants

Characteristics	Total study cohort (*N* = 3051)	Participants with GDM (*N* = 313)	Participants without GDM (*N* = 2738)	*p* value^a^
Age (years), mean (SD)	32.0 (5.0)	32.3 (4.8)	31.9 (5.0)	0.18
< 30	934 (30.6)	89 (28.4)	845 (30.9)	0.85
30 - <35	1200 (39.3)	126 (40.3)	1074 (39.2)	
35 - <40	721 (23.6)	77 (24.6)	644 (23.5)	
≥ 40	196 (6.4)	21 (6.7)	175 (6.4)	
Maternal ethnicity				< 0.001*
European	1449 (47.5)	87 (27.8)	1362 (49.7)	
Māori	157 (5.1)	12 (3.8)	145 (5.3)	
Pacific peoples	334 (11.0)	39 (12.5)	295 (10.8)	
Asian	936 (30.7)	151 (48.2)	785 (28.7)	
Other	175 (5.7)	24 (7.7)	151 (5.5)	
BMI (kg/m²), mean (SD)	27.4 (6.1)	29.2 (6.8)	27.2 (6.0)	< 0.001*
< 25	1266 (41.5)	85 (27.2)	1181 (43.1)	< 0.001*
25.0 - <30	1018 (33.4)	112 (35.8)	906 (33.1)	
≥ 30	767 (25.1)	116 (37.1)	651 (23.8)	
Parity				0.42
0	1524 (49.9)	167 (53.3)	1357 (49.6)	
1	909 (29.8)	85 (27.2)	824 (30.1)	
≥ 2	618 (20.3)	61 (19.5)	557 (20.3)	
Any previous perinatal death^b^ (N = 1601)	55 (3.4)	7 (4.5)	48 (3.3)	0.44
Gestational age at OGTT (weeks), median (IQR)	27.3 (26.3–28.3)	27.4 (26.3–28.4)	27.1 (26.3–28.1)	0.03*
Family history of diabetes	1069 (35.0)	160 (51.1)	909 (33.2)	< 0.001*
OGTT results (mmol/L), median (IQR)				
Fasting	4.3 (4.1–4.6)	4.9 (4.4–5.4)	4.3 (4.1–4.5)	< 0.001*
1-hour postprandial	7.5 (6.3–8.5)	10.1 (9.2–11.0)	7.4 (6.1–8.1)	< 0.001*
2-hour postprandial	6.1 (5.2–7.1)	8.7 (7.2–9.6)	5.9 (5.1–6.8)	< 0.001*

^a^ comparison between women with GDM and women without GDM

^b^Among women with previous pregnancy of 20 weeks’ or more

Data presented as number (%), unless otherwise indicated. p-values < 0.05 denoted with asterisks (*)

BMI– body mass index; IQR– Interquartile range; OGTT– oral glucose tolerance test; SD– standard deviation

### Prevalence and risk for poor perinatal mental health

At baseline (median 27.3 weeks’ gestation), a higher proportion of participants with GDM [43/313 (14%)] compared to participants without GDM [321/2718 (11.8%)] reported symptoms reflecting risk for anxiety, although this was not statistically significant [RR:1.17 (95% CI 0.87, 1.57)] (Table 2). The proportion of participants at risk of depression was similarly not different between groups, although the GDM group reported higher mean EPDS scores [MD:0.63 (0.12,1.14)]. In the SF-36 domains relating to mental health, participants with GDM had lower scores for social functioning, but the overall MCS measures were not significantly different between the two groups. In the SF-36 domains relating to physical health, the overall PCS measures were lower (worse) in the GDM group [PCS MD: -2.97 (-3.98, -1.97)], as were physical functioning, role physical, bodily pain, and general health.

At 36 weeks’ gestation, the prevalence of risk for anxiety and depression were not significantly different between the two groups and both groups had similar STAI and EPDS scores. There was no overall difference between groups in HRQoL, although participants with GDM had lower SF-36 general health scores [MD: -3.58 (-5.69, 1.47)] and higher vitality scores [MD: 3.08 (1.34, 4.81)] compared to participants with no GDM.

At 6 months postpartum, the risk for anxiety and depression was not different between the two groups and both groups had similar STAI and EPDS scores. However, SF-36 vitality and the overall MCS measure were higher (better) among the GDM group [MCS MD: 1.28 (0.25, 2.30)], whereas physical functioning, role physical, bodily pain, general health, and social functioning, and the overall PCS measure were all lower in the GDM group [PCS MD: -2.99 (-3.90, -2.07)].

**Table 2:** Comparison of perinatal mental health between GDM group and no GDM group

Outcome	GDM		No GDM			
	*N*	*n* (%) or mean (SD)	*N*	*n* (%) or mean (SD)	Relative risk or mean difference (95% CI)	*p* value
**Time of study enrolment (baseline)**						
Any mental disorder ^a^	313	70 (22.4)	2738	516 (18.9)	1.19 (0.95, 1.48)	0.13
Anxiety (STAI > 15)	308	43 (14.0)	2718	321 (11.8)	1.17 (0.87, 1.57)	0.29
STAI score	308	10.6 (3.4)	2718	10.3 (3.3)	0.38 (-0.01, 0.77)	0.06
Depression (EPDS > 12)	308	36 (11.7)	2714	257 (9.5)	1.23 (0.89, 1.71)	0.22
EPDS score	308	7.1 (4.4)	2714	6.5 (4.3)	0.63 (0.12, 1.14)	0.01*
Poor mental HRQoL (MCS < 40)	308	40 (13.0)	2714	304 (11.2)	1.16 (0.85, 1.57)	0.36
SF-36 scores:						
Physical functioning	309	65.6 (21.5)	2728	72.8 (20.6)	-7.33 (-9.67, -4.89)	< 0.001*
Role physical	312	58.4 (41.2)	2729	66.4 (38.0)	-7.97 (-12.46, -3.47)	< 0.001*
Bodily pain	312	66.3 (20.5)	2738	70.7 (20.1)	-4.50 (-6.86, -2.13)	< 0.001*
General health	313	72.2 (18.4)	2733	75.2 (17.0)	-2.94 (-4.94, -0.94)	0.004*
Vitality	313	56.5 (13.7)	2733	55.2 (13.6)	1.22 (-0.37, 2.81)	0.13
Social functioning	314	77.1 (21.1)	2739	81.7 (20.6)	-4.53 (-6.94, -2.12)	< 0.001*
Role emotional	312	82.6 (32.2)	2728	85.6 (29.4)	-3.03 (-6.50, 0.45)	0.09
Mental health	312	69.7 (11.2)	2737	70.3 (10.8)	-0.67 (-1.94, 0.60)	0.30
PCS	308	43.6 (8.6)	2714	46.6 (8.5)	-3.01 (-4.02, -2.00)	< 0.001*
MCS	308	49.4 (7.7)	2714	49.0 (7.3)	0.38 (-0.48, 1.24)	0.39
**36 weeks’ gestation**						
Any mental disorder ^a^	298	72 (24.2)	2590	573 (22.1)	1.09 (0.88, 1.35)	0.42
Anxiety (STAI > 15)	295	47 (15.9)	2586	365 (14.1)	1.13 (0.86, 1.49)	0.39
STAI score	296	10.7 (3.4)	2586	10.7 (3.4)	0.07 (-0.34, 0.47)	0.74
Depression (EPDS > 12)	296	35 (11.0)	2588	282 (10.9)	1.08 (0.78, 1.50)	0.64
EPDS score	317	6.7 (4.5)	2588	6.6 (4.5)	0.16 (-0.38, 0.71)	0.55
Poor mental HRQoL (MCS < 40)	296	38 (12.8)	2590	321 (12.4)	1.03 (0.75, 1.41)	0.84
SF-36 scores:						
Physical functioning	296	58.2 (20.7)	2590	58.9 (21.8)	-0.78 (-3.38, 1.83)	0.56
Role physical	297	46.7 (41.5)	2590	42.4 (39.6)	4.32 (-0.46, 9.10)	0.08
Bodily pain	297	61.6 (22.1)	2590	60.8 (20.4)	0.82 (-1.65, 3.30)	0.51
General health	297	70.7 (19.1)	2590	74.3 (17.4)	-3.58 (-5.69, -1.47)	0.001*
Vitality	297	54.2 (14.8)	2590	51.2 (14.4)	3.08 (1.34, 4.81)	< 0.001*
Social functioning	297	75.5 (21.3)	2590	75.8 (21.5)	-0.27 (-2.85, 2.31)	0.84
Role emotional	297	79.5 (35.4)	2590	79.4 (35.3)	0.04 (-4.20, 4.29)	0.99
Mental health	297	69.7 (11.8)	2590	69.6 (11.3)	0.03 (-1.33, 1.40)	0.96
PCS	296	40.2 (9.0)	2590	40.1 (9.1)	0.10 (-0.99, 1.19)	0.86
MCS	296	49.9 (7.9)	2590	49.7 (7.8)	0.18 (-0.77, 1.12)	0.71
**6 months postpartum**						
Any mental disorder ^a^	302	71 (23.5)	1780	417 (23.4)	1.00 (0.80, 1.25)	0.97
Anxiety (STAI > 15)	301	45 (14.9)	1777	219 (12.3)	1.21 (0.90, 1.63)	0.21
STAI score	301	10.6 (3.3)	1777	10.4 (3.3)	0.22 (-0.19, 0.63)	0.29
Depression (EPDS > 12)	300	23 (7.7)	1779	161 (9.0)	0.84 (0.55, 1.28)	0.43
EPDS score	300	6.0 (4.4)	1779	5.9 (4.5)	0.13 (-0.41, 0.68)	0.63
Poor mental HRQoL (MCS < 40)	298	46 (15.4)	1777	331 (18.6)	0.83 (0.62, 1.10)	0.18
SF-36 scores:						
Physical functioning	300	81.8 (22.8)	1779	89.2 (16.4)	-7.44 (-9.58, -5.30)	< 0.001*
Role physical	300	81.1 (31.9)	1778	85.6 (29.2)	-4.53 (-8.16, -0.91)	0.01*
Bodily pain	301	75.3 (21.9)	1780	79.6 (20.0)	-4.26 (-6.73, -1.78)	< 0.001*
General health	300	73.6 (19.0)	1780	76.6 (17.1)	-2.99 (-5.12, -0.86)	0.006*
Vitality	300	59.0 (14.2)	1780	55.4 (14.5)	3.52 (1.74, 5.29)	< 0.001*
Social functioning	301	81.8 (21.1)	1780	85.5 (19.6)	-3.67 (-6.10, -1.25)	0.003*
Role emotional	300	85.2 (30.1)	1778	84.3 (31.2)	0.92 (-2.88, 4.72)	0.63
Mental health	300	70.2 (12.0)	1780	70.1 (11.6)	0.09 (-1.33, 1.52)	0.90
PCS	298	50.6 (8.6)	1777	53.6 (7.3)	-2.99 (-3.90, -2.07)	< 0.001*
MCS	298	47.7 (8.0)	1777	46.4 (8.4)	1.28 (0.25, 2.30)	0.01*

^a^Any of anxiety (STAI > 15), vulnerability to depression (EPDS > 12) or poor mental health-related quality of life (MCS < 40)

EPDS- Edinburgh Postnatal Depression Scale; HRQoL– health-related quality of life; MCS- mental component score; PCS– physical component score; SF-36–36-Item Short-Form General Health Survey; STAI– Spielberger State-Trait Anxiety Inventory

*p*-values < 0.05 denoted with asterisks (*)

### Changes in mental health measures over time

**Fig. 1:**
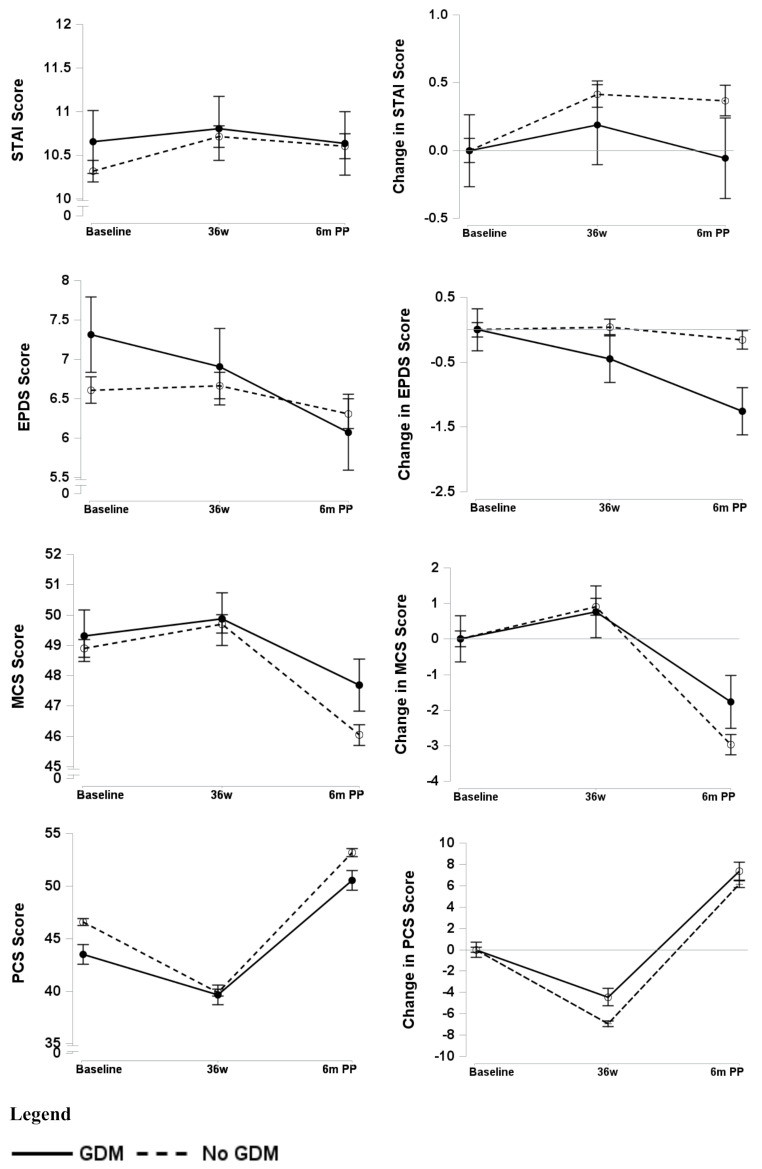
Comparison of mental health scores and changes over time between participants with GDM and those without GDM. GDM; gestational diabetes mellitus, 36w; 36 weeks gestation, 6 m PP; 6 months postpartum

Participants in both groups reported a similar trajectory for mean STAI scores over time (Table 3; Fig. 1). After adjustment for baseline scores, participants in both groups reported slightly better STAI scores at 36 weeks’ gestation and a slight decline in scores at 6 months postpartum, with no significant time by group interaction effects.

For EPDS scores, participants with GDM reported a mean decline from baseline of 0.5 score at 36 weeks and around 1.5 score at 6 months postpartum, whereas participants with no GDM reported no significant changes from baseline at 36 weeks’ gestation and 6 months postpartum (interaction p-value 0.003).

The direction of change in the overall MCS and PCS measures was similar for participants in both groups. For MCS, both groups experienced an improvement from baseline of around one score at 36 weeks’ gestation. At 6 months postpartum, there was a decline in MCS scores, with the GDM group experiencing less of a decline (better functioning) compared to the no GDM group (interaction p-value = 0.03). For PCS, both groups reported a decline in mean scores at 36 weeks’ gestation but participants with GDM reported less of a decline (better physical functioning) compared to those without GDM. At 6 months postpartum, mean PCS scores had improved for both groups, but the GDM group reported less improvement (worse functioning) from 36 weeks’ gestation compared to the no GDM group (interaction p-value = < 0.001).

**Table 3:** Longitudinal changes in mental health measures: Group by time interactions

	GDM	No GDM			
	Mean scores or *n*/*N* (%) (95% CI)	Mean scores or *n*/*N* (%) (95% CI)	Mean difference or relative risk (95% CI)	*p* value	*p* value for interaction
**Anxiety**					
**STAI score**					
Time of enrolment	10.6 (10.3, 11.0)	10.3 (10.2, 10.4)	0.34 (-0.04, 0.71)	0.08	0.29
36 weeks’ gestation	10.8 (10.4, 11.2)	10.7 (10.6, 10.8)	0.08 (-0.31, 0.47)	0.68	
6 months postpartum	10.6 (10.3, 11.0)	10.6 (10.4, 10.7)	0.06 (-0.34, 0.45)	0.77	
**STAI > 15**					
Time of enrolment (*N* = 3026)	43/308 (13.9) (10.1, 17.8)	321/2718 (11.8) (10.6, 13.0)	1.18 (0.88, 1.58)	0.28	0.94
36 weeks’ gestation (*N* = 2882)	47/296 (15.9) (11.7, 20.0)	365/2586 (14.1) (12.8, 15.5)	1.13 (0.85, 1.49)	0.41	
6 months postpartum (*N* = 2078)	45/302 (14.9) (10.9, 18.9)	219/1777 (12.3) (10.8, 13.9)	1.21 (0.90, 1.63)	0.21	
**Depression**					
**EPDS score**					
Time of enrolment	7.3 (6.9, 7.8)	6.6 (6.5, 6.8)	0.71 (0.22, 1.20)	0.005*	0.003*
36 weeks’ gestation	6.9 (6.4, 7.4)	6.7 (6.5, 6.8)	0.23 (-0.28, 0.75)	0.37	
6 months postpartum	6.1 (5.6, 6.6)	6.3 (6.1, 6.5)	-0.19 (-0.73, 0.34)	0.48	
**EPDS > 12**					
Time of enrolment (*N* = 3022)	36/308 (11.6) (8.1, 15.2)	257/2714 (9.5) (8.4, 10.6)	1.23 (0.89, 1.71)	0.22	0.38
36 weeks’ gestation (*N* = 2885)	35/297 (11.8) (8.1, 15.4)	282/2588 (10.9) (9.7, 12.1)	1.08 (0.78, 1.50)	0.64	
6 months postpartum (*N* = 2079)	23/301 (7.6) (4.6, 10.6)	161/1779 (9.1) (7.8, 10.4)	0.84 (0.55, 1.28)	0.43	
**Health related quality of life**					
**SF-36 scores**					
**Physical functioning**					
Time of enrolment	65.3 (63.0, 67.6)	72.7 (71.9, 73.4)	-7.37 (-9.75, -4.98)	< 0.001*	< 0.001*
36 weeks’ gestation	56.9 (54.5, 59.4)	58.3 (57.5, 59.1)	-1.36 (-3.92, 1.20)	0.30	
6 months postpartum	81.3 (79.4, 83.3)	88.3 (87.5, 89.0)	-6.95 (-9.06, -4.84)	< 0.001*	
**Role physical**					
Time of enrolment	57.9 (53.7, 62.1)	66.2 (64.7, 67.6)	-8.29 (-12.72, -3.85)	< 0.001*	< 0.001*
36 weeks’ gestation	45.4 (40.9, 49.9)	42.0 (40.5, 43.6)	3.36 (-1.34, 8.07)	0.16	
6 months postpartum	81.1 (77.7, 84.4)	85.2 (83.8, 86.5)	-4.11 (-7.71, -0.51)	0.02*	
**Bodily pain**					
Time of enrolment	66.4 (64.2, 68.6)	70.7 (69.9, 71.4)	-4.30 (-6.62, -1.98)	< 0.001*	0.001*
36 weeks’ gestation	60.8 (58.6, 63.1)	60.4 (59.6, 61.2)	0.45 (-1.99, 2.84)	0.73	
6 months postpartum	75.2 (73.0, 77.5)	78.8 (77.9, 79.7)	-3.58 (-6.03, -1.14)	0.004*	
**General health**					
Time of enrolment	71.9 (70.1, 73.7)	74.9 (74.3, 75.6)	-3.01 (-4.93, -1.09)	0.002*	0.12
36 weeks’ gestation	70.1 (68.2, 72.0)	73.9 (73.3, 74.6)	-3.83 (-5.85, -1.83)	< 0.001*	
6 months postpartum	73.6 (71.8, 75.5)	75.4 (74.7, 76.1)	-1.76 (-3.78, 0.27)	0.09	
**Vitality**					
Time of enrolment	56.5 (55.1, 58.0)	55.3 (54.8, 55.8)	1.22 (-0.32, 2.75)	0.12	0.005*
36 weeks’ gestation	54.4 (52.8, 55.9)	51.3 (50.8, 51.8)	3.09 (1.44, 4.74)	< 0.001*	
6 months postpartum	59.3 (57.7, 60.9)	55.5 (54.9, 56.1)	3.78 (2.07, 5.49)	< 0.001*	
**Social functioning**					
Time of enrolment	76.6 (74.4, 78.9)	81.2 (80.5, 82.0)	-4.60 (-6.96, -2.23)	< 0.001*	0.01*
36 weeks’ gestation	74.6 (72.2, 76.9)	75.3 (74.5, 76.1)	-0.75 (-3.26, 1.76)	0.56	
6 months postpartum	81.7 (79.5, 83.9)	84.2 (83.3, 85.1)	-2.53 (-4.92, -0.15)	0.04*	
**Role emotional**					
Time of enrolment	82.6 (79.3, 85.8)	85.3 (84.2, 86.4)	-2.72 (-6.16, 0.72)	0.12	0.15
36 weeks’ gestation	78.3 (74.4, 82.3)	79.0 (77.7, 80.4)	-0.70 (-4.88, 3.49)	0.74	
6 months postpartum	85.1 (81.6, 88.6)	83.3 (81.9, 84.7)	1.80 (-1.97, 5.64)	0.35	
**Mental health**					
Time of enrolment	69.6 (68.4, 70.7)	70.1 (69.8, 70.5)	-0.62 (-1.85, 0.61)	0.32	0.13
36 weeks’ gestation	69.6 (68.3, 70.8)	69.5 (69.1, 69.9)	0.06 (-1.24, 1.37)	0.92	
6 months postpartum	70.1 (68.8, 71.4)	69.3 (68.8, 69.8)	0.77 (-0.63, 2.16)	0.28	
**PCS**					
Time of enrolment	43.5 (42.6, 44.4)	46.6 (46.3, 46.9)	-3.07 (-4.05, -2.09)	< 0.001*	< 0.001*
36 weeks’ gestation	39.6 (38.6, 40.6)	39.8 (39.5, 40.2)	-0.20 (-1.27, 0.87)	0.71	
6 months postpartum	50.5 (49.7, 51.3)	53.3 (52.9, 53.6)	-2.75 (-3.65, -1.85)	< 0.001*	
**MCS**					
Time of enrolment	49.3 (48.5, 50.1)	48.9 (48.6, 49.2)	0.41 (-0.43, 1.25)	0.34	0.03*
36 weeks’ gestation	49.9 (49.0, 50.7)	49.7 (49.4, 50.0)	0.19 (-0.73, 1.10)	0.69	
6 months postpartum	47.7 (46.7, 48.6)	46.1 (45.7, 46.4)	1.63 (0.62, 2.64)	0.002*	
**MCS < 40**					
Time of enrolment (*N* = 3022)	40/308 (12.9) (9.2, 16.7)	304/2714 (11.2) (10.0, 12.4)	1.16 (0.85, 1.57)	0.36	0.27
36 weeks’ gestation (*N* = 2886)	38/297 (12.8) (9.0, 16.6)	321/2590 (12.4) (11.1, 13.7)	1.03 (0.75, 1.41)	0.84	
6 months postpartum (*N* = 2075)	46/299 (15.4) (11.3, 19.5)	331/1777 (18.6) (16.8, 20.4)	0.83 (0.62, 1.10)	0.18	

EPDS- Edinburgh Postnatal Depression Scale; HRQoL– health-related quality of life; MCS- mental component score; PCS– physical component score; SF-36–36-Item Short-Form General Health Survey; STAI– Spielberger State-Trait Anxiety Inventory

p-values < 0.05 denoted with asterisks (*)

## Discussion

In this multi-ethnic cohort of more than 3000 participants, we found no difference in risk for anxiety or depression among participants with GDM compared to participants without GDM in late gestation and 6 months after birth. However, participants in the GDM group reported higher EPDS scores at study enrolment and these declined over time compared to participants in the no GDM group. Both physical and mental HRQoL differed between the two groups. In late gestation, participants in the GDM group reported better physical HRQoL, and in the postnatal period, better mental HRQoL but worse physical HRQoL compared to participants in the no GDM group.

### Anxiety

Around one in eight women self-reported symptoms of risk for anxiety in both groups over the assessed time periods. Studies which have reported a higher prevalence of anxiety in women with GDM have suggested that the perception of high-risk pregnancy and the stressful treatment regimen associated with GDM management contributes to this increase (Hui et al. 2014; Ouyang et al. 2021). However, a meta-analysis of observational studies reported no difference in the odds for anxiety in women with GDM compared to women without GDM (Delanerolle et al. 2021), and a recent study assessing prevalence of antenatal anxiety symptoms in a small cohort of women with GDM reported that anxiety symptomatology was not significantly associated with glycemic control (Munda et al. 2021). These findings are consistent with our finding of similar anxiety prevalence in participants with and without GDM.

The prevalence of anxiety symptoms may also depend on the time of assessment. GDM diagnosis may be associated with some reactive anxiety during pregnancy which settles in late gestation and in the postpartum period due to reassurance from care (Daniells et al. 2003). The changes in anxiety scores over time in our study support this, with the GDM group having higher scores at baseline but decreasing by 36 weeks.

### Depression

Around one in ten participants reported symptoms of vulnerability to depression in both groups over the assessed time periods, an estimate which is consistent with previous findings in New Zealand (Underwood et al. 2017), and other high-income countries (Woody et al. 2017). No difference was found in the risk for vulnerability to depression between the two groups. However, participants with GDM reported a one EPDS score higher than participants without GDM at baseline. Shared biological mechanisms such as dysregulation of the hypothalamic-pituitary axis (HPA)(Zhao et al. 2022) and psychosocial risk factors including stress associated with treatment adherence have been implicated in the link between diabetes and depressive symptoms (Holt et al. 2014). Among women with GDM, HPA dysregulation results in elevated concentrations of cortisol, a hormone implicated in depression (Keller et al. 2017). Additionally, recent qualitative evidence suggests that around the time of GDM diagnosis, limited knowledge and misconceptions about the diagnosis affects women’s psychological well-being negatively (Benton et al. 2024). Our finding of a higher EPDS score at baseline may be linked to a higher proportion of participants with GDM being obese (BMI ≥ 30 kg/m^2^). Obesity in the antenatal period has been reported to increase the risk for depressive symptoms. A 1.05 increase in EPDS score per kg/m^2^ increase in BMI was reported among a pregnancy cohort in Australia (Sominsky et al. 2023). Additionally, the higher proportion of women with GDM reporting a family history of diabetes may be contributing to the higher EPDS score at baseline. Half of the participants with GDM in our study reported a family history of diabetes compared to one in three participants without GDM. Some evidence suggests family history predisposes to insulin resistance (Ong et al. 2022), which has been positively associated with depressive symptoms (Kan et al. 2013). However, the extent to which this may have contributed to the higher baseline EPDS scores in participants with GDM in our study is difficult to determine as we did not assess insulin resistance.

The decline in EPDS scores in late gestation and up to 6 months after birth in participants with GDM is consistent with evidence that the increased care and access to information associated with GDM care results in reduced depressive symptoms in the postpartum period (Crowther et al. 2005).

### Health-related quality of life

Assessing health related quality of life in the perinatal period is increasingly important to understand the physical and mental changes associated with this period, and hence may play an important role in clinical decision making to provide relevant and appropriate care in the perinatal period (Wu et al. 2021).

In our study, quality of life related to mental health and functioning was similar for participants in both groups in the antenatal period and declined six months after birth, but with better mental HRQoL in participants with GDM compared to those without. Other studies have reported inconsistent results. A small cohort study in Italy reported worse mental HRQoL two months after the birth in participants who were diagnosed with GDM compared to controls, and suggested that this was due to a persisting sense of poor health due to possible knowledge about long-term risks associated with the diagnosis (Dalfrà et al. 2012). Other studies have reported no difference in psychological quality of life comparing pregnant women who had GDM to pregnant women without complications (Mautner et al. 2009; Halkoaho et al. 2010). In our study, the finding of better mental HRQoL among participants with GDM could be due to the additional pregnancy and postpartum care they receive. This is consistent with randomized clinical trial evidence which reported improved health-related quality of life (including both physical and mental health domains) three months postpartum for participants who received treatment for GDM compared to those who received routine antenatal care (Crowther et al. 2005).

In the postpartum period, exercise and increased access to information and support has also been linked to improved quality of life and self-rated health (Haas et al. 2005; Campolong et al. 2018). Due to the increased risk for future cardiometabolic disorders, it is recommended that women whose pregnancies are complicated by GDM continue to receive lifestyle management in the postnatal period (American Diabetes Association 2022). This extra care and access to information could be contributing to the findings in this cohort as well. Those not diagnosed with GDM are less likely to continue with healthy lifestyle choices when there is less access to information and support (Bagherzadeh et al. 2021).

Participants with GDM reported a lower physical HRQoL at baseline compared to those with no GDM. This could be due to factors such as obesity, and increasing age which can predispose pregnant women to developing GDM and are also associated with poor physical HRQoL during pregnancy (Emmanuel and Sun 2014; Lagadec et al. 2018; Lee et al. 2018). Participants in the GDM group were older and a higher proportion were obese compared to participants in the no GDM group. Additionally, the lower physical HRQoL among participants with GDM could be related to the higher EPDS scores found at baseline in this group. Setse et al. found a longitudinal association between depressive symptoms and poor physical functioning in the first and second trimesters among pregnant women and suggested that this association could be bidirectional, with depression predisposing to lesser HRQoL, and the perception of a poor quality of life contributing to depressive symptoms via shared psychosocial risk factors (Setse et al. 2009), emphasizing the comorbid nature of poor perinatal mental health among women with GDM.

For all participants, physical HRQoL declined in late pregnancy and improved six months after birth. This decline in late gestation has been associated with pregnancy symptoms including indigestion and poor sleep quality (Haas et al. 2005). However, participants with GDM experienced less decline in overall physical HRQoL in late gestation than those without GDM, possibly related to the emphasis on lifestyle management in GDM care which may promote efforts in better physical functioning among participants in the GDM group. This is consistent with evidence from other studies (Dalfrà et al. 2012).

In the postpartum period, physical quality of life improved in both groups, likely due to physical recovery from childbirth (Emmanuel and Sun 2014). Although participants with GDM reported lower mean scores for physical HRQoL at 6 months postpartum, both groups experienced a similar improvement in physical HRQoL from late gestation to the postpartum period. Hence, this difference in scores was likely due to the difference in baseline scores.

### Strengths and limitations

The main strength of this study is use of prospectively collected longitudinal data from a large multi-ethnic trial, making our results generalisable to the New Zealand population and ensuring appropriate comparison groups of women with and without GDM. We were also able to examine different aspects of mental health in the antenatal and postnatal periods and undertake longitudinal analysis of changes over time.

However, the assessment tools used in screening for perinatal disorders, although validated for this population, are not the gold standard to diagnose these disorders.

The lack of data on pre-pregnancy mental health status of participants in our study, a factor which can predict perinatal mental disorders (Guintivano et al. 2018), may also be a limitation to interpretation of our results. Additionally, we did not adjust for possible confounders relating to low socioeconomic status and lack of social support as data on these factors were not collected as part of the study. Furthermore, no adjustment for multiplicity was performed, hence, significant p-values should be interpreted with caution.

## Conclusion

In this longitudinal analysis involving a large cohort of pregnant women, participants who were diagnosed and treated for GDM had similar risk of anxiety and vulnerability to depression compared with participants who were not diagnosed with GDM. However, participants with GDM reported better mental health-related quality of life but poorer physical health-related quality of life in the postnatal period. Future studies should explore the determinants of perinatal mental health among pregnant women to assess if they differ by GDM status.

Our findings are consistent with evidence suggesting that treatment of GDM may improve women’s quality of life and reduce their vulnerability to depression into the postnatal period, providing reassurance for health professionals. However, additional support around the perinatal period may be needed to ensure optimal physical functioning for women with GDM.

## Data Availability

The data sets generated and/or analysed during the current study are not publicly available as ethical approval for this study did not include sharing of individual data. The corresponding author is the guarantor of this work and, as such, had full access to all the data in the study and takes responsibility for the integrity of the data and the accuracy of the data analysis.

## References

[CR1] Ahmadinezhad GS et al (2024) ‘Association between postpartum depression and breastfeeding self-efficacy in mothers: a systematic review and meta-analysis’, *BMC Pregnancy and Childbirth*, 24(1), pp. 1–10. Available at: 10.1186/s12884-024-06465-4 10.1186/s12884-024-06465-4 PMC11015580 38609849

[CR2] Al-abri K, Edge D, Armitage CJ (2023) ‘Prevalence and correlates of perinatal depression’, *Social Psychiatry and Psychiatric Epidemiology*, 1, p. 3. Available at: 10.1007/s00127-022-02386-9 10.1007/s00127-022-02386-9 PMC9842219 36646936

[CR3] American Diabetes Association (2022) ‘15. Management of Diabetes in Pregnancy: Standards of Medical Care in Diabetes—2022’, *Diabetes Care*, 45, pp. S232–S243. Available at: 10.2337/dc22-S015 10.2337/dc22-S015 34964864

[CR4] Arafa A, Dong JY (2019) ‘Gestational diabetes and risk of postpartum depressive symptoms: A meta-analysis of cohort studies’, *Journal of Affective Disorders*, 253, pp. 312–316. Available at: 10.1016/j.jad.2019.05.001 10.1016/j.jad.2019.05.001 31078830

[CR5] Azami M et al (2019) ‘The association between gestational diabetes and postpartum depression: A systematic review and meta-analysis’, *Diabetes Research and Clinical Practice*, 149, pp. 147–155. Available at: 10.1016/j.diabres.2019.01.034 10.1016/j.diabres.2019.01.034 30735772

[CR6] Bagherzadeh R et al (2021) ‘Pregnancy; an opportunity to return to a healthy lifestyle: a qualitative study’, *BMC Pregnancy and Childbirth*, 21(1), pp. 1–11. Available at: 10.1186/s12884-021-04213-6 10.1186/s12884-021-04213-6 PMC8569967 34740317

[CR7] Bauer A, Knapp M, Parsonage M (2016) ‘Lifetime costs of perinatal anxiety and depression’, *Journal of Affective Disorders*, 192, pp. 83–90. Available at: 10.1016/J.JAD.2015.12.005 10.1016/j.jad.2015.12.005 26707352

[CR8] Benton M et al (2024) ‘The (un)controlled body: A grounded theory analysis to conceptualise stigma for women with gestational diabetes mellitus’, *Journal of Health Psychology*, pp. 1–16. Available at: 10.1177/13591053241241863 10.1177/13591053241241863 PMC11977814 38628073

[CR9] Campolong K et al (2018) ‘The association of exercise during pregnancy with trimester-specific and postpartum quality of life and depressive symptoms in a cohort of healthy pregnant women’, *Archives of Women’s Mental Health*, 21(2), pp. 215–224. Available at: 10.1007/s00737-017-0783-0 10.1007/s00737-017-0783-0 29067551

[CR10] Court H, Greenland K, Margrain TH (2010) ‘Measuring patient anxiety in primary care: Rasch analysis of the 6-item Spielberger state anxiety scale’, *Value in Health*, 13(6), pp. 813–819. Available at: 10.1111/j.1524-4733.2010.00758.x 10.1111/j.1524-4733.2010.00758.x 20561315

[CR11] Cox JL, Holden JM, Sagovsky R (1987) ‘Detection of postnatal depression: Development of the 10-item Edinburgh Postnatal Depression scale’, *British Journal of Psychiatry*, 150, pp. 782–786. Available at: 10.1192/bjp.150.6.782 10.1192/bjp.150.6.782 3651732

[CR12] Crowther CA et al (2005) ‘Effect of Treatment of Gestational Diabetes Mellitus on Pregnancy Outcomes’, *New England Journal of Medicine*, 352(24), pp. 2477–2486. Available at: 10.1056/nejmoa042973 10.1056/NEJMoa042973 15951574

[CR13] Crowther CA et al (2022) ‘Lower versus Higher Glycemic Criteria for Diagnosis of Gestational Diabetes’, *New England Journal of Medicine*, 387(7), pp. 587–598. Available at: 10.1056/nejmoa2204091 10.1056/NEJMoa2204091 36070709

[CR14] Dadi AF et al (2020) ‘Global burden of antenatal depression and its association with adverse birth outcomes: An umbrella review’, *BMC Public Health*, 20(1). Available at: 10.1186/s12889-020-8293-9 10.1186/s12889-020-8293-9 PMC7001252 32019560

[CR15] Dalfrà MG et al (2012) ‘Quality of life in pregnancy and post-partum: A study in diabetic patients’, *Quality of Life Research*, 21(2), pp. 291–298. Available at: 10.1007/s11136-011-9940-5 10.1007/s11136-011-9940-5 21633879

[CR16] Daniells S et al (2003) ‘Gestational Diabetes Mellitus Is a diagnosis associated with an increase in maternal anxiety and stress in the short and intermediate term?’, *Diabetes Care*, 26(2), pp. 385–389. Available at: 10.2337/DIACARE.26.2.385 10.2337/diacare.26.2.385 12547867

[CR17] Delanerolle G et al (2021) ‘A systematic review and meta-analysis of gestational diabetes mellitus and mental health among BAME populations’, *eClinicalMedicine*, 38. Available at: 10.1016/j.eclinm.2021.101016 10.1016/j.eclinm.2021.101016 PMC8283332 34308317

[CR18] Dennis CL, Falah-Hassani K, Shiri R (2017) ‘Prevalence of antenatal and postnatal anxiety: Systematic review and meta-analysis’, *British Journal of Psychiatry*, 210(5), pp. 315–323. Available at: 10.1192/bjp.bp.116.187179 10.1192/bjp.bp.116.187179 28302701

[CR19] Emmanuel EN, Sun J (2014) ‘Health related quality of life across the perinatal period among Australian women’, *Journal of Clinical Nursing*, 23(11–12), pp. 1611–1619. Available at: 10.1111/jocn.12265 10.1111/jocn.12265 23750859

[CR20] George A et al (2013) ‘Anxiety symptoms and coping strategies in the perinatal period’, *BMC Pregnancy and Childbirth*, 13(233). Available at: 10.1186/1471-2393-13-233 10.1186/1471-2393-13-233 PMC3867672 24330429

[CR21] Guintivano J, Manuck T, Meltzer-Brody S (2018) ‘Predictors of Postpartum Depression: A comprehensive review of the last decade of evidence’, *Clinical obstetrics and gynecology*, 61(3), p. 591. Available at: 10.1097/GRF.0000000000000368 10.1097/GRF.0000000000000368 PMC6059965 29596076

[CR22] Haas JS et al (2005) ‘Changes in the health status of women during and after pregnancy’, *Journal of General Internal Medicine*, 20(1), pp. 45–51. Available at: 10.1111/j.1525-1497.2004.40097.x 10.1111/j.1525-1497.2004.40097.x PMC1490030 15693927

[CR23] Halkoaho A et al (2010) ‘Does gestational diabetes affect women’s health-related quality of life after delivery?’, *European Journal of Obstetrics and Gynecology and Reproductive Biology*, 148(1), pp. 40–43. Available at: 10.1016/j.ejogrb.2009.09.025 10.1016/j.ejogrb.2009.09.025 19883969

[CR24] Holt RIG et al (2014) ‘NIDDK international conference report on diabetes and depression: Current understanding and future directions’, *Diabetes Care*, 37(8), pp. 2067–2077. Available at: 10.2337/dc13-2134 10.2337/dc13-2134 PMC4113168 25061135

[CR25] Hui AL et al (2014) ‘Stress and anxiety in women with gestational diabetes during dietary management’, *The Diabetes Educator*, 40(5), pp. 668–677. Available at: 10.1177/0145721714535991 10.1177/0145721714535991 24874692

[CR26] Kan C et al (2013) ‘A systematic review and meta-analysis of the association between depression and insulin resistance’, *Diabetes Care*, 36(2), pp. 480–489. Available at: 10.2337/dc12-1442 10.2337/dc12-1442 PMC3554272 23349152

[CR27] Keller J et al (2017) ‘HPA axis in major depression: Cortisol, clinical symptomatology and genetic variation predict cognition’, *Molecular Psychiatry*, 22(4), pp. 527–536. Available at: 10.1038/mp.2016.120 10.1038/mp.2016.120 PMC5313380 27528460

[CR28] Lagadec N et al (2018) ‘Factors influencing the quality of life of pregnant women: A systematic review’, *BMC Pregnancy and Childbirth*, 18(1), pp. 1–14. Available at: 10.1186/s12884-018-2087-4 10.1186/s12884-018-2087-4 PMC6251086 30470200

[CR29] Lee KW et al (2018) ‘Prevalence and risk factors of gestational diabetes mellitus in Asia: a systematic review and meta-analysis’, *BMC Pregnancy and Childbirth*, 18(1), pp. 1–20. Available at: 10.1186/S12884-018-2131-4 10.1186/s12884-018-2131-4 PMC6295048 30547769

[CR30] Lee KW et al (2020) ‘Neonatal outcomes and its association among gestational diabetes mellitus with and without depression, anxiety and stress symptoms in Malaysia: A cross-sectional study’, *Midwifery*, 81, p. 102586. Available at: 10.1016/j.midw.2019.102586 10.1016/j.midw.2019.102586 31830674

[CR31] Levis B et al (2020) ‘Accuracy of the Edinburgh Postnatal Depression Scale (EPDS) for screening to detect major depression among pregnant and postpartum women: Systematic review and meta-analysis of individual participant data’, *BMJ*. Available at: 10.1136/bmj.m4022 10.1136/bmj.m4022 PMC7656313 33177069

[CR32] Luca DL et al (2020) ‘Financial Toll of Untreated Perinatal Mood and Anxiety Disorders Among 2017 Births in the United States’, *American journal of public health*, 110(6), pp. 888–896. Available at: 10.2105/AJPH.2020.305619 10.2105/AJPH.2020.305619 PMC7204436 32298167

[CR33] Marchetti D et al (2017) ‘Quality of life in women with gestational diabetes mellitus: A systematic review’, *Journal of Diabetes Research*. Hindawi Limited, pp. 7058082–7058082. Available at: 10.1155/2017/7058082 10.1155/2017/7058082 PMC5343261 28326332

[CR34] Mautner E et al (2009) ‘Quality of life outcomes in pregnancy and postpartum complicated by hypertensive disorders, gestational diabetes, and preterm birth’, *Journal of Psychosomatic Obstetrics and Gynecology*, 30(4), pp. 231–237. Available at: 10.3109/01674820903254757 10.3109/01674820903254757 19845493

[CR35] Mennes M et al (2006) ‘Long-term cognitive sequelae of antenatal maternal anxiety: involvement of the orbitofrontal cortex’, *Neuroscience and Biobehavioral Reviews*, 30(8), pp. 1078–1086. Available at: 10.1016/j.neubiorev.2006.04.003 10.1016/j.neubiorev.2006.04.003 16780948

[CR36] Munda A, Fekonja U, Pongrac Barlovič D (2021) ‘Prevalence of depressive and anxiety symptoms in women with gestational diabetes: a longitudinal cohort study’, *Acta Diabetologica*, 58(8), pp. 1091–1100. Available at: 10.1007/s00592-021-01706-w 10.1007/s00592-021-01706-w 33772371

[CR38] O’Connor TG, Heron J, Glover V (2002) ‘Antenatal Anxiety Predicts Child Behavioral/Emotional Problems Independently of Postnatal Depression’, *Journal of the American Academy of Child & Adolescent Psychiatry*, 41(12), pp. 1470–1477. Available at: 10.1097/00004583-200212000-00019 10.1097/00004583-200212000-00019 12447034

[CR37] O’Connor TG et al (2003) ‘Maternal antenatal anxiety and behavioural/emotional problems in children: a test of a programming hypothesis’, *Journal of Child Psychology and Psychiatry*, 44(7), pp. 1025–1036. Available at: 10.1111/1469-7610.00187 10.1111/1469-7610.00187 14531585

[CR39] Ong SL et al (2022) ‘Family history of diabetes moderates metabolic depression endophenotypes in overweight/obese adults’, *Journal of Psychiatric Research*, 151(February), pp. 583–589. Available at: 10.1016/j.jpsychires.2022.05.018 10.1016/j.jpsychires.2022.05.018 35636036

[CR40] Ouyang H et al (2021) ‘Associations between Gestational Diabetes and Anxiety or Depression: A Systematic Review’, *Journal of Diabetes Research*, 2021. Available at: 10.1155/2021/9959779 10.1155/2021/9959779 PMC8337159 34368368

[CR41] Packer CH et al (2021) ‘Increased rates of adverse perinatal outcomes in women with gestational diabetes and depression’, *Journal of Maternal-Fetal and Neonatal Medicine*, 34(23), pp. 3862–3866. Available at: 10.1080/14767058.2019.1701647 10.1080/14767058.2019.1701647 31851552

[CR42] Pfoh ER et al (2016) ‘The SF-36 offers a strong measure of mental health symptoms in survivors of acute respiratory failure: A tri-national analysis’, *Annals of the American Thoracic Society*, 13(8), pp. 1343–1350. Available at: 10.1513/AnnalsATS.201510-705OC 10.1513/AnnalsATS.201510-705OC PMC5021072 27111262

[CR43] Plows JF et al (2018) ‘The pathophysiology of gestational diabetes mellitus’, *International Journal of Molecular Sciences*, 19(11). Available at: 10.3390/ijms19113342 10.3390/ijms19113342 PMC6274679 30373146

[CR44] Riggin L (2020) ‘Association between gestational diabetes and mental illness’, *Canadian Journal of Diabetes*, 44(6), pp. 566–571. Available at: 10.1016/j.jcjd.2020.06.014 10.1016/j.jcjd.2020.06.014 32792108

[CR45] Rusner M, Berg M, Begley C (2016) ‘Bipolar disorder in pregnancy and childbirth: A systematic review of outcomes’, *BMC Pregnancy and Childbirth*, 16(1). Available at: 10.1186/s12884-016-1127-1 10.1186/s12884-016-1127-1 PMC5084442 27793111

[CR46] Setse R et al (2009) ‘Longitudinal study of depressive symptoms and health-related quality of life during pregnancy and after delivery: The health status in pregnancy (HIP) study’, *Maternal and Child Health Journal*, 13(5), pp. 577–587. Available at: 10.1007/s10995-008-0392-7 10.1007/s10995-008-0392-7 18931832

[CR47] Shen Y et al (2020) ‘Association of gestational diabetes mellitus with adverse pregnancy outcomes: our experience and meta-analysis’, *International Journal of Diabetes in Developing Countries*, 40(3), pp. 357–370. Available at: 10.1007/S13410-020-00802-X

[CR48] Sominsky L et al (2023) ‘Pre-pregnancy obesity is associated with greater systemic inflammation and increased risk of antenatal depression’, *Brain, Behavior, and Immunity*, 113(June), pp. 189–202. Available at: 10.1016/j.bbi.2023.07.005 10.1016/j.bbi.2023.07.005 37437818

[CR49] The Ministry of Health New Zealand (1999) *Taking the Pulse - The 1996/97 New Zealand Health Survey - SF-36 data analysis*. Wellington. Available at: https://www.health.govt.nz/publication/taking-pulse-1996-97-new-zealand-health-survey-sf-36-data-analysis (Accessed: 29 July 2022)

[CR50] Underwood L et al (2017) ‘A Longitudinal Study of Pre-pregnancy and Pregnancy Risk Factors Associated with Antenatal and Postnatal Symptoms of Depression: Evidence from Growing Up in New Zealand’, *Maternal and Child Health Journal*, 21(4), pp. 915–931. Available at: 10.1007/s10995-016-2191-x 10.1007/s10995-016-2191-x 27837388

[CR52] Ware JE (2000) ‘SF-36 Health Survey update’, *Spine*, 25(24), pp. 3130–3139. Available at: 10.1097/00007632-200012150-00008 10.1097/00007632-200012150-00008 11124729

[CR51] Ware J, MA, K. and, Keller SD (1993) ‘SF-36 Physical and Mental Health Summary Scales: a User’s Manual’, 8, pp. 23–28

[CR53] Ware JE, Sherbourn CD (1992) The MOS 36-Item short-Form Health Survey (SF-36): conceptual framework and item selection. Med Care 30(6):473–483 1593914

[CR54] Wilson CA et al (2020) ‘Is there an increased risk of perinatal mental disorder in women with gestational diabetes? A systematic review and meta-analysis’, *Diabetic Medicine*, 37(4), pp. 602–622. Available at: 10.1111/dme.14170 10.1111/dme.14170 PMC7154542 31693201

[CR55] Woody CA et al (2017) ‘A systematic review and meta-regression of the prevalence and incidence of perinatal depression’, *Journal of Affective Disorders*, 219(May), pp. 86–92. Available at: 10.1016/j.jad.2017.05.003 10.1016/j.jad.2017.05.003 28531848

[CR56] World Health Organization (1999) *Definition, diagnosis and classification of diabetes mellitus and its complications. Part 1: Diagnosis and classification of diabetes mellitus*. Geneva

[CR57] Wu H et al (2021) ‘Health-related quality of life in different trimesters during pregnancy’, *Health and Quality of Life Outcomes*, 19(1), pp. 1–11. Available at: 10.1186/s12955-021-01811-y 10.1186/s12955-021-01811-y PMC8296584 34289867

[CR58] Zhao R et al (2022) ‘Effect of Gestational Diabetes on Postpartum Depression-like Behavior in Rats and Its Mechanism’, *Nutrients*, 14(6), pp. 1–19. Available at: 10.3390/nu14061229 10.3390/nu14061229 PMC8953401 35334886

